# Lower limb control and mobility following exercise training

**DOI:** 10.1186/1743-0003-9-15

**Published:** 2012-02-15

**Authors:** Sukwon Kim, Thurmon Lockhart

**Affiliations:** 1 Department of Physical Education, Chonbuk National University, Jeonju-City, South Korea; 2 Grado Department of Industrial and Systems Engineering, Virginia Tech, Blacksburg, VA 24060

**Keywords:** Joint Stiffness, Limb Mobility, Exercise, Stability, Falls

## Abstract

The objective of the present study was to evaluate the effects of 8-week balance or weight training on ankle joint stiffness and limb stability for older adults, furthermore, on outcomes of slips while walking. Eighteen older adults volunteered for the study and randomly were assigned to the three groups, such as, weight, balance, or control group. While walking on a walking track, three-dimensional posture data were sampled and ankle joint stiffness and limb stability were computed to evaluate the effects of training. 2 (pre and post) × 3 (weight, balance, and control) × 2 (dominant and non-dominant legs) mixed factor repeated ANOVA was performed. The results indicated that only balance training group showed an improvement in joint stiffness and both the training groups showed improvements in limb stability. Also, fall frequency results suggested that joint stiffness and limb stability had an effect on the likelihood of slip-induced falls. In conclusion, training can facilitate improvements in joint and limb control mechanism for older adults contributing to an improvement in the likelihood of slip-induced falls.

## Introduction

Without sufficient single limb strength, a person cannot transport the body safely and efficiently across terrain because the single limb support or transition takes a significant portion of normal walking. Particularly, a production of adequate ankle joint torque is needed to move the whole body forward safely [1,2]. However, it becomes more difficult as ankle muscle strength and ankle joint flexibility continue to deteriorate with advancing age [3,4]

During single limb support, the control applied by the limb is depicted by the joint torque produced for any given joint angle [1,5]. This relationship (the gradient of the torque-angle graph) is named joint stiffness [1,5,6]. Joint stiffness, particularly at the ankle, increases with advancing age [7] and is accompanied with decreases in ankle strength [8]. Degradations in ankle muscle strength or range of motion may result in an increase in ankle joint stiffness leading to unstable gait patterns [7,9-11]. This may increase the likelihood of falls among older adults [2,12].

An individual possessing steeper ankle stiffness shows unstable postural balance [7]. The contractile capability of the plantar flexor muscles signifies ankle muscle strength [13], which plays a major role in adjusting the whole body center-of-mass when the postural balance is disturbed such as a slip [14]. Thus, a general decrease in ankle muscle strength with advancing age may interfere with e a person's ability to recover postural balance and may increase the potential for a slip-induced fall. However, little about the effects of ankle joint stiffness on slips has been known. Therefore, the present study aimed to evaluate the role of ankle joint stiffness on the risk of falling among older adults.

In addition to ankle joint stiffness, the present study evaluated if limb stability had an effect on the likelihood of a slip-induced fall. Limb stability or control, which has been characterized by variability of gait parameters, may be one of key factors when assessing the likelihood of a slip-induced fall [9,15]. In the previous studies, fall-prone individuals showed larger variability of gait characteristics [9,16]. Typically, the previous studies [9,16] looked at the stride-to-stride variability such as stride length, double support time, swing time, and velocity. However, no study has identified limb stability, while transitioning on a single limb whereas the other limb is in swing phase, as a key factor for increasing the likelihood of slips and falls. The present study hypothesized that improvements in ankle joint stiffness and limb stability (i.e. single limb support) due to exercise training would result in a reduction in the likelihood of slips and falls for older adults.

*Intervention *Proper sensorimotor control of lower extremity is critical when a body attempts to sustain mobility and joint stability [11,17-19]. Either balance or weight training improves muscle strength and sensorimotor function in lower extremity in relation to gait or balance [20-24]. The present study used both the training regimens to improve sensorimotor control of lower extremity.

## Methods

### Participants

Each participant completed an inform consent form approved by the University's Internal Review Board (IRB). Participants were excluded from the study if they indicated any physical problems (i.e. hip, knee, ankle problems); a questionnaire was used as an initial screening tool. They were recruited from the local community. Participants who had no history of the formal weight and balance exercises in the past 6 months were identified as eligible participants for this study. It would not be matter if they were involved in any form of exercises such as walking, running, swimming, dancing, gardening, tennis, golf, etc. They were eligible for participating for the present study if they did not have any formal weight and balance exercises in the past 6 months. Be more specific, they were eligible for the study if their physical activities did not involve any intensions that particularly targeted weight lifting and balance control. In addition, participants were allowed to participate in the present study if the length of their exercises targeting to improve muscle strength as well as balance did not exceed more than total 30 minutes a week (i.e. for example, weight and balance exercises for 2 time a week for 10 minutes for a session (total 20 minutes) would not be considered as the formal weight and balance training). The investigator interviewed the elderly for screening for the study before evaluating their gait characteristics and other parameters.

To ensure that the weight or balance training group did not engage to any other exercises or physical activities during 8 weeks, the investigator monitored their daily activity. During all training sessions, the investigator (trainer) ensured that all participants in training groups followed the exercise routines correctly. Individuals in control group were not allowed to engage in any form of exercises or in any form of physical activities; to ensure that, the investigator interviewed the elderly in the control group during the social meetings.

Total of 24 healthy older individuals (2 males and 22 females) participated in the study at the beginning. By the week 4, five participants dropped out of the program leaving 7 in balance, 6 people in weight, and 6 people in control groups. All 19 people lasted for 8 weeks. However, in order to balance out the number of participants for each group, only 18 individuals' data were evaluated.

Power analysis was perform to satisfy Type I error of 0.05 and Type II error of < 0.35 (Power > 0.65) using JMP statistical packages (SAS Institute Inc. Cary, NC, USA). Lower Power (> 0.65) was chosen as the acceptable power since the analysis was performed on a 6-week strength training (Knight and Kamen, 2001) not an 8-week strength training.

### Balance and Weight Training

#### Balance training

For the first week, in order for all the volunteers to be familiar with the exercise routines (table 1), they were instructed to perform the exercises provided in the instructional manual of Stability Trainer (Thera-Band^®^, 1245 Home Avenue, Akron, OH 44310, see the following link for instruction and product detail, http://www.thera-band.com/store/products.php?ProductID=24, http://www.thera-band.com/instructions.php) on firm surfaces such as floors. During 2^nd ^week, all volunteers were evaluated if they were able to perform the exercises on green stability trainer (intermediate challenge level). If an individual was not able to perform the exercise routines safely and accurately on the stability trainer, she/he continued to perform the exercise on firm surface until she/he was able to perform the exercises safely on the green stability trainer. In addition, blue stability trainers (advanced challenge level) were introduced if an individual performed exercises perfectly and confidently on the green stability trainer. Among 6 participants, only 2 progressed to perform the exercises on blue stability trainer. No upper body exercise was introduced for this particular balance training.

**Table 1:** Exercise Regimen for Balance Training with Stability Trainer

Exercises	Exercise Descriptions
Bilateral balance with squat	Standing shoulder apart on the foams with hands on waist, participants bring hip down as low as possible like sitting on a chair while pausing the position for 3 seconds and bring hip up while resting for 3 seconds. Participants repeat the exercise for 10-12 times.

Bilateral calf raises	Standing on the foams with hands on waist, participants raise ankles as high as possible while holding the position for 2 seconds and bring ankle down while resting for 3 seconds. Participants repeat the exercise for 10 times.

Unilateral balance	Participants stand on one leg on the foam. Standing on the foams with hands on waist, participants keep balance as long as possible. Participants repeat the exercise 3 times for each leg

Unilateral calf raises	Participants stand on one leg on the foam. Standing on the foams with hands on waist, participants raise ankle as high as possible. Participants repeat the exercise 10 times for each leg

Unilateral balance with leg backward kick	Participants stand on one leg on the foam. Standing on the foam with hands on waist, participants bring back non-supported leg as much as possible without loosing balance and hold the position for 3 seconds. Participants repeat 10 times for each leg.

Unilateral balance with hip flexion	Participants stand on one leg on the foam. Standing on the foam with hands on waist, participants bring forward non-supported leg as much as possible without losing balance and hold the position for 3 seconds. Participants repeat 10 times for each leg

Unilateral balance with knee flexion	Participants stand on one leg on the foam. Standing on the foam with hands on waist, participants flex their non-supported knee about 90° without losing balance and hold the position for 3 seconds. Participants repeat 10 times for each leg.

Kick (abduction and adduction)	Abduction: participants stand on one leg on the foam. Standing on the foam with hands on waist, participants abduct their non-supported leg as much as possible without losing balance and hold the position for 3 seconds. Participants repeat 10 times for each leg. Adduction: participants stand on one leg on the foam. Standing on the foam with hands on waist, participants adduct their non-supported leg as much as possible without losing balance and hold the position for 3 seconds. Participants repeat 10 times for each leg.

Sit-to-stand	While feet are resting on the foam, participants stand up without losing balance from a chair with no help from hands.

Forward reach	While standing on the foam, participants reach an object at their waist height and hold the position for 3 seconds. It is repeated for 10 times.

Lunge	While standing on the floor, step on the foam and lower the body as much as possible. Participants hold the position for 3 seconds and repeat the exercise for 10 times.

#### Weight training

For weight training, periodized strength training than non-periodized strength training was implemented as it was proven to be more effective in gaining strength [25]. Two different hypertrophy phases was introduced for 5 weeks; 3 sets of 10 repetitions with 50% of maximum exertion for 2 weeks and 3 sets of 10 repetitions for 70% of maximum exertion for 3 weeks. Strength phase lasted for the last 3 weeks; 3 sets of 7 repetitions with 85% of maximum exertion.

Weight training was performed in NS-4000 home gym model (Nautilus^®^, Vancouver, Washington 98684).

All volunteers performed six weight lifting exercises for legs.

1) Seated leg press: target muscle (quadriceps), Synergists (Gluteus Maximus, Adductor Magnus, and Soleus)

2) Calf press: target muscle (gastrocnemius), Synergists (Soleus)

3) Leg curl: target muscle (hamstring), Synergists (gastrocnemius and sartorius)

4) Leg extension: target muscle (quadriceps), Synergists (gleuteus maximus, adductor magnus, soleus),

5) Hip abduction and adduction: target muscle (gluteus medius, minimus, and maximus), synergists (pectineus, gracilis)

6) Hip extension: target muscle (gluteus maximus) synergists (gluteus medius and minimus)

A 45-60 second resting period was given between each set and a 150-180 second resting period was given between exercises. No upper body exercise was introduced for this particular weight training.

#### Apparatus

**Figure 1:**
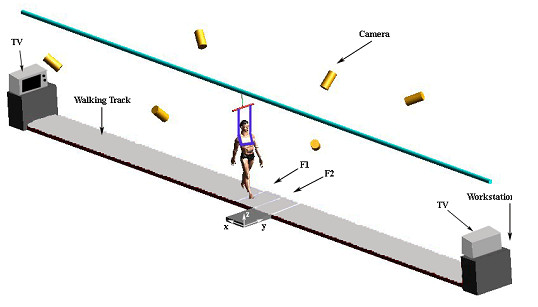
**Field layout of the experimental set-up including; Fall Arresting System, Infra-red cameras (6), Two force plate (F1 and F2), and workstations**. X, Y, and Z = global references for force and position.

Walking trials were conducted on a walking track (20 m), which was elevated 15 cm above the floor surface (Figure 1). A six-camera ProReflex system (Qualysis) was used to collect three-dimensional posture data of participants as they walked over the test floor surface. Kinematic data were sampled and recorded at 120 Hz.

A set of 24 markers were placed on participants' anthropometric landmarks (base of second toe(2 markers), malleolus(4), epicondyle (4), greater trochater(2), base of first phalange of third finger(2), styloid process of ulna(2), lateral epicondyle of humerus(2), greater tubercle (2), acromion(2), and anterior portion of temporal bone(2). They were instructed to walk straight and to look forward while walking at their preferred walking speed. Participants' cadence was continuously monitored within a subsequent 20 minute session in order to ensure that their natural walking speed was consistent throughout the session. After ensuring that the preferred walking speeds were consistent, participants' natural posture was collected. Exact same procedure was performed before and after training.

Ankle joint centers were defined as the midpoints between medial and lateral malleolus markers. The global coordinate system was constructed based upon the fixed laboratory coordinate which is identical with the coordinate space utilized in the motion capture system. The global coordinate system was then transformed into the local coordinate system using the Gram-Schmidt orthogonalization process [26] as described in Liu and Lockhart [10].

*Dynamic ankle stiffness *(JS) during walking was measured as the change in ankle moment through the period of second rocker during gait cycle [5] for detail in definition of dynamic ankle stiffness). The slope of the curve over this period was computed using a polynomial regression strategy [5].

**Figure 2:**
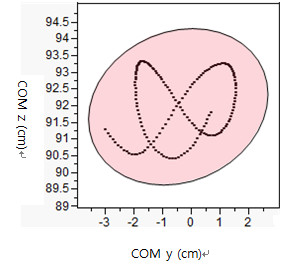
**Illustration of Factor Analysis in Limb Stability**.

*Center of Mass *in (x, y, z) was calculated by averaging all the center of mass of the 14 segments (left and right feet, left and right shanks, left and right thighs, trunk, left and right hands, left and right lower arms, left and right upper arms, head). COM position of the entire mass is the sum of all the segment (part) mass and com products divided by the total mass (1).

(1) $$x_{cm}=\frac{\overset{N}{\underset{i=1}{\sum }}m_{i}x_{i}}{M}\;y_{cm}=\frac{\overset{N}{\underset{i=1}{\sum }}m_{i}y_{i}}{M}\;z_{cm}=\frac{\overset{N}{\underset{i=1}{\sum }}m_{i}z_{i}}{M}$$

where cm is the center of mass,

m_i _is the mass of segment,

x_i_, y_i _, z_i _is the distance from segment end,

M is the total mass (weight of a person),

Then, *limb stability (LS, cm^2^) *was evaluated using factor analysis (multivariate analysis) which often was used as a structure detection method. COM in y direction (COM y) and COM in z direction (COM z) during heel-contact to toe-off of each leg were identified as two separate factors (Figure 2). An eigenvalue on each factor (COM y and COM z) was identified as the variance on each factor. Since there were only two factors, two factors account for 100 percent of the variance of these two sets (COM y and z) of data. These two eigenvalues were used to create ellipse area [27]. Larger an ellipse generally indicated larger variability of COM in medio-lateral and/or longitudinal directions. The larger variability suggested larger sway of the body during walking. Instable limb support or control may contribute to larger sway of the body while walking.

In order to identify '*FALLS*', Slip distance, sliding heel velocity, the whole body COM velocity and motion pictures were considered [28]. To be considered as a fall, the slip distance must exceed 10 cm, and peak sliding heel velocity must exceed the whole body COM velocity while slipping [28]. In addition, videos for each slip trial of the participant were analyzed to assess if an actual fall had occurred. All of above three conditions had to be met to be considered as a fall.

### Data Analysis

JS and LS for all groups (weight, balance, and control groups) were evaluated at pre- and post-training. 2 (Time; pre and post) × 3 (Group; weight, balance, and control) × 2 (Leg; dominant and non-dominant) mixed factor repeated measure ANOVA was performed by utilizing the JMP statistical packages (SAS Institute Inc. Cary, NC, USA) to evaluate the effects of training on each group. Training was a between-subjects factor and time was within-subjects factors. Student's t (*post hoc*) was used to perform multiple comparisons when main effects were found significant. The bivariate analysis was performed to see the relationships between joint stiffness and limb stability at pre- and post- training stages. The results were considered as statistically significant when p ≤ 0.05.

## Results

### Joint Stiffness (JS)

**Figure 3:**
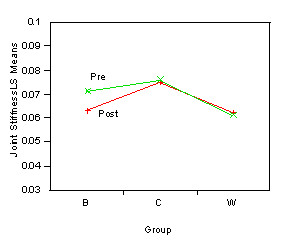
**Two-way interaction plot of Time × Group in Joint Stiffness**.

There was no main effect. There was an interaction effect of Time*Group (p = 0.03). Only the balance training group exhibited a reduction in joint stiffness after 8 weeks (Figure 3 and student's t-test). However, the data indicated that JS for balance training group did not differ from JS for weight training group.

### Limb Stability (LS)

**Figure 4:**
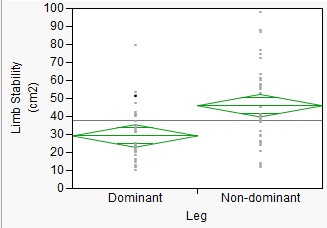
**ANOVA comparison of Limb Stability by Leg**.

**Figure 5:**
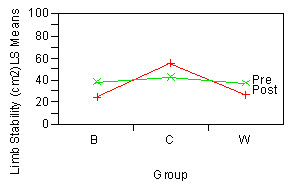
**Two-way interaction plot of Time × Group in Limb Stability**.

LS (p = 0.0003) was different between dominant leg (29.24 cm^2^) and non-dominant leg (46.06 cm^2^) (Figure 4). There were interaction effects of Time*Group (p = 0.003). Figure 5 and student's t suggested that training groups exhibited better limb stability, whereas, control group did not show a difference after 8 weeks.

### Isokinetic Plantarflexor strength

The results (table 2) indicated that isokinetic plantarflexion ankle strength improved after training, mainly, in balance as well as weight training groups.

**Table 2:** ANOVA of isokinetic ankle plantarflexion strengths at various speeds

		Weight (N = 6)	Balance (N = 6)	Control (N = 6)	P (Time)	P (Group× Time)
D 30 Ex	Pre	38.3 ± 17.5	34.5 ± 8.5	22.7 ± 7.6	0.003	0.003
	Post	40.0 ± 9.2	45.7 ± 12.1	23.7 ± 6.0		

D 90 Ex	Pre	27.3 ± 17.3	30.0 ± 9.2	17.5 ± 6.9	0.01	0.05
	Post	40.2 ± 8.1	35.5 ± 11.8	17.0 ± 4.3		

N 30 Ex	Pre	35.3 ± 9.6	37.2 ± 12.2	24.3 ± 6.9	0.0006	0.07
	Post	49.3 ± 9.0	45.0 ± 12.0	26.7 ± 10.0		

N 90 Ex	Pre	29.2 ± 11.1	27.3 ± 12.1	17.3 ± 8.0	0.0006	0.02
	Post	38.5 ± 10.9	34.2 ± 10.9	17.3 ± 6.7		

* D = dominant leg, N = non-dominant leg, Ex = plantarflexion

### Correlations between limb stability and joint stiffness

**Figure 6:**
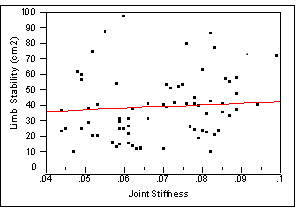
**Correlation plot of Limb Stability by Joint Stiffness**.

**Figure 7:**
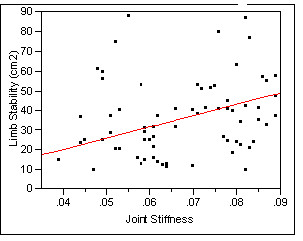
**Correlation plot of Limb Stability by Joint Stiffness**.

The results (Figure 6) indicated that, at pre-training stage, scores of limb stability was not correlated to joint stiffness evaluated at pre-training stage. However, after training, the results (Figure 7) indicated that scores of limb stability was statistically correlated to joint stiffness (Rsquare = 0.13, p = 0.03). This could suggest that limb stability improved as joint stiffness became smaller due to exercise training.

### The likelihood of slip-induced fall

In balance training group or weight training group, 4 individuals in each group who fell in the pre-training stage recovered from slips and 2 individuals in each group who recovered from slips in the pre-training stage recovered from slips after 8 week training. In control group, 5 individuals who fell in the pre-training stage fell again and 1 individual who recovered from a slip again recovered after 8 weeks. These results with consistent gait characteristics suggested that older individuals with training showed more chance to recover from slips.

## Discussion

The objective of the present study was to assess the effects of 8-week balance or weight training on ankle joint stiffness and limb stability and to determine if joint stiffness and/or limb stability were key indicators in association with the likelihood of falls.

*Dynamic ankle stiffness *(JS) during walking is expressed as the ratio of ankle joint torque to the ankle range of motion (i.e. joint angles) through the period of second rocker during gait cycle [5]. Previous studies [3,4] suggested that a production of ankle joint torque played a major role in moving the whole body forward safely and efficiently across terrain. The elderly could not produce adequate ankle joint torque as rapidly as the younger adults while moving the body [3,4]. Furthermore, the risk of falling in the elderly increased as ankle joint flexibility was reduced [2]. JS was suggested to increase as ankle joint flexibility continued to decrease with advancing age [7]. The results from the present study indicated that only balance training group exhibited decrements in JS due to training. However, after training, JS (0.063) of balance group became similar with JS (0.062) of weight training group although JS (0.075) of control group differed from JS of balance or weight group. In addition, JS of weight training group at pre-training stage was 0.061. These results may suggest that the weight training group's ankle flexibility was already better than balance group's ankle flexibility. By these results, authors speculates that JS between 0.062 and 0.063 would be good for the elderly during walking. Many studies evaluated JS and suggested that it was an important indicator for pathological gait, aging gait, postural instability, and gait instability. However, no study has clearly stated the usefulness of this measure such that this measure could be used for intervention strategies. Therefore, studies in regard to assessments of optimal range of JS for the elderly while walking should be performed to, further, advocate importance of joint stiffness measure.

Limb stability had not clearly been investigated although many studies evaluated gait variability, strength, or joint stiffness to explain limb stability. These parameters only represented a portion of limb stability. These parameters could not entirely represent dynamic limb stability. Older adults are known to adapt to safer gait in order to avoid falling [16,29]. Still, falls are the major concern for them because older adults still falls even through they adapt to safer gait. This suggests that safer gait does not entirely means "Safe" while walking. Studies [9,16] reported that older adults exhibited unstable joints or gait in comparison to younger adults and, further, suggested that these local instabilities seen among the elderly contributed to falls among the elderly. The present study desired to test if these gait instability could be improved by exercise training and, in addition, to evaluate asymmetry of gait stability between the legs. The results indicated that limb stability improved only in training group. Limb stability in the present study was a fundamental measure which represented the entire sum of gait instability or variability for the elderly. Limb stability measure in the present study indicated an outcome of all the combined factors that maybe contribute to gait instability or variability while walking.

In the present study, improvements in LS was correlated to decreases in JS at post-training stage, whereas, LS was not correlated with JS at pre-training stage. These results suggested that JS undoubtedly provided important information about limb control as suggested by Salsich and Mueller [30]. Improved flexibility, seen in smoother rate of increments of joint moments throughout the range of motion, as well as improved limb stability in the present study suggested that limb control was mainly derived from contractile capability of ankle plantarflexor muscles [30,31] which became stronger after training. The ability for ankle plantarflexor muscles to control or stabilize the ankle joint throughout forward progression of COM represents the limb control as well as limb stability. Better limb stability most likely indicates smaller variability in the medio-lateral COM. Therefore, improved limb stability stems from smoother sinusoidal progression of COM which, in turn, suggests smoother rate of changes in joint moments throughout changes in joint angles. Improvements in limb stability in training group further supported that training facilitated improvements in limb control mechanisms among older adults.

In agreement with previous suggestions (Allard et al., 1996; Barr et al., 1987; Law, 1987; Rosenrot et al., 1980; Stefanyshyn et al., 1980), LS of dominant leg was different from LS of non-dominant leg (Figure 4). The result indicated that dominant leg had better LS in comparison to non-dominant leg. Limb dominance can take place when one side is preferred to the other side in activities such as kicking. To date, no study has suggested relationships between limb dominance and limb stability although many studies [32-35] have suggested relationship between limb dominance and gait asymmetry in the lower limbs. The results from the present study suggested that progressing body forward over dominant leg was much safer indicated by its smaller variability when comparing to non-dominant leg. Furthermore, results in Time × Group interaction indicated that LS improved due to training. These results suggested that limb dominance played a role in gait stability and the effects of limb dominance could be minimized by exercise training.

## Competing interests

The authors declare that they have no competing interests.

## Authors' contributions

SK and TL have made substantial contributions to conception and design, interpretation of data, and SK has been involved in drafting and revising the manuscript. SK has been involved in acquisition of data and analysis of data. All authors read and approved the final manuscript.
